# Supplementary figures and images for: Comparative specificity and sensitivity of NS1-based serological assays for the detection of flavivirus immune response
Source: PLoS Negl Trop Dis. 2020 Jan 29;14(1):e0008039. doi: 10.1371/journal.pntd.0008039 (PMC7010293; doi:10.1371/journal.pntd.0008039)

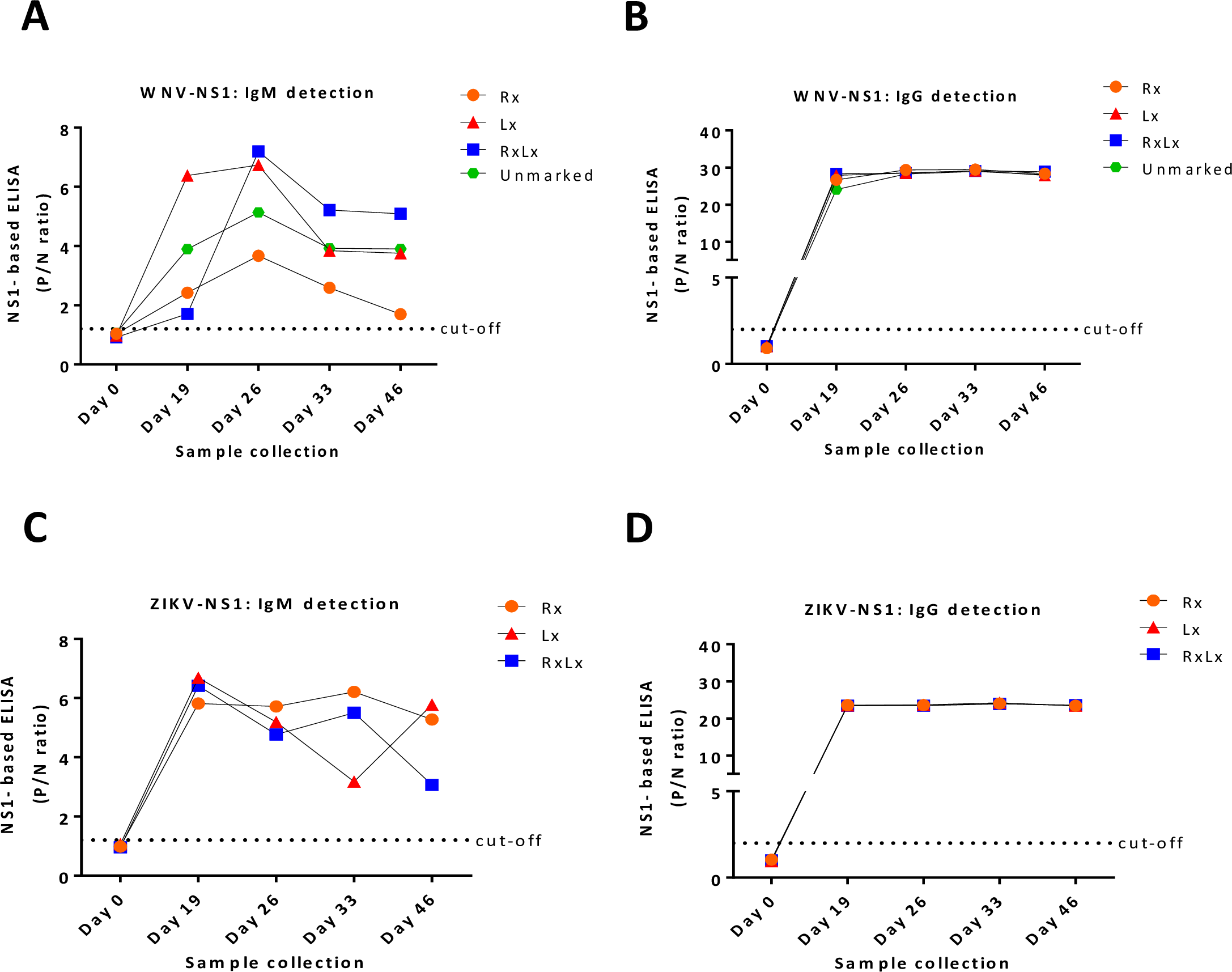

Supplement: S1 Fig — A and B) Detection of IgM/IgG antibodies from 4 different immunized mice with WNV NS1 V5-tagged construct. C and D) Detection of IgM/IgG antibodies from 3 different immunized mice with ZIKV NS1 V5-tagged construct. Plates were coated with purified WNV-rNS1 and ZIKV-rNS1 antigens for the detection of antibodies from mice immunized with WNV-NS1 and ZIKV-NS1, respectively. * each ELISA result includes the average of two biological replicates. (TIF) [file pntd.0008039.s001.tif]

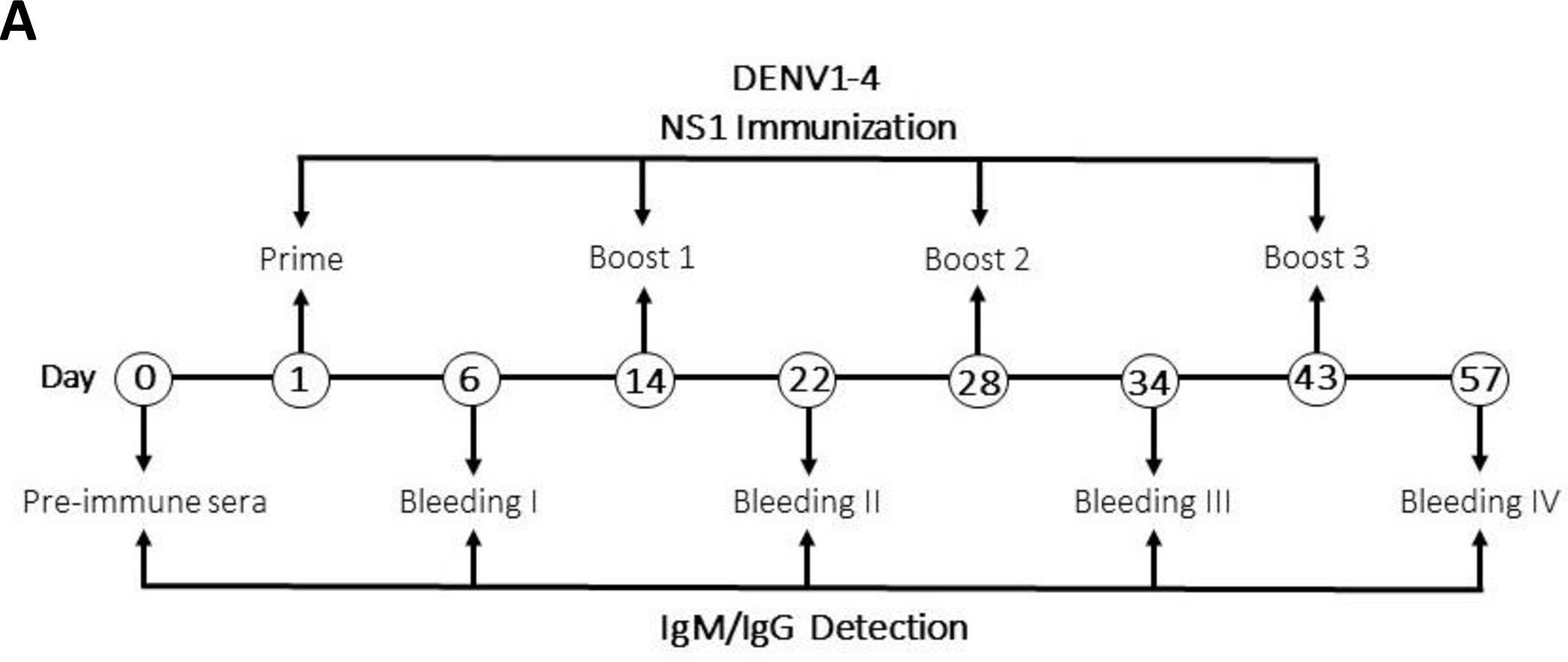

Supplement: S2 Fig — 4 different BALB/c mice for each DENV serotype (DENV-1, DENV-2, DENV-3, and DENV-4) were immunized with specific NS1 V5-tagged plasmids by gene gun technology. The day when the prime-boost immunization was performed, and sera samples were collected for IgM/IgG detection are indicated in numbers. (TIF) [file pntd.0008039.s002.tif]

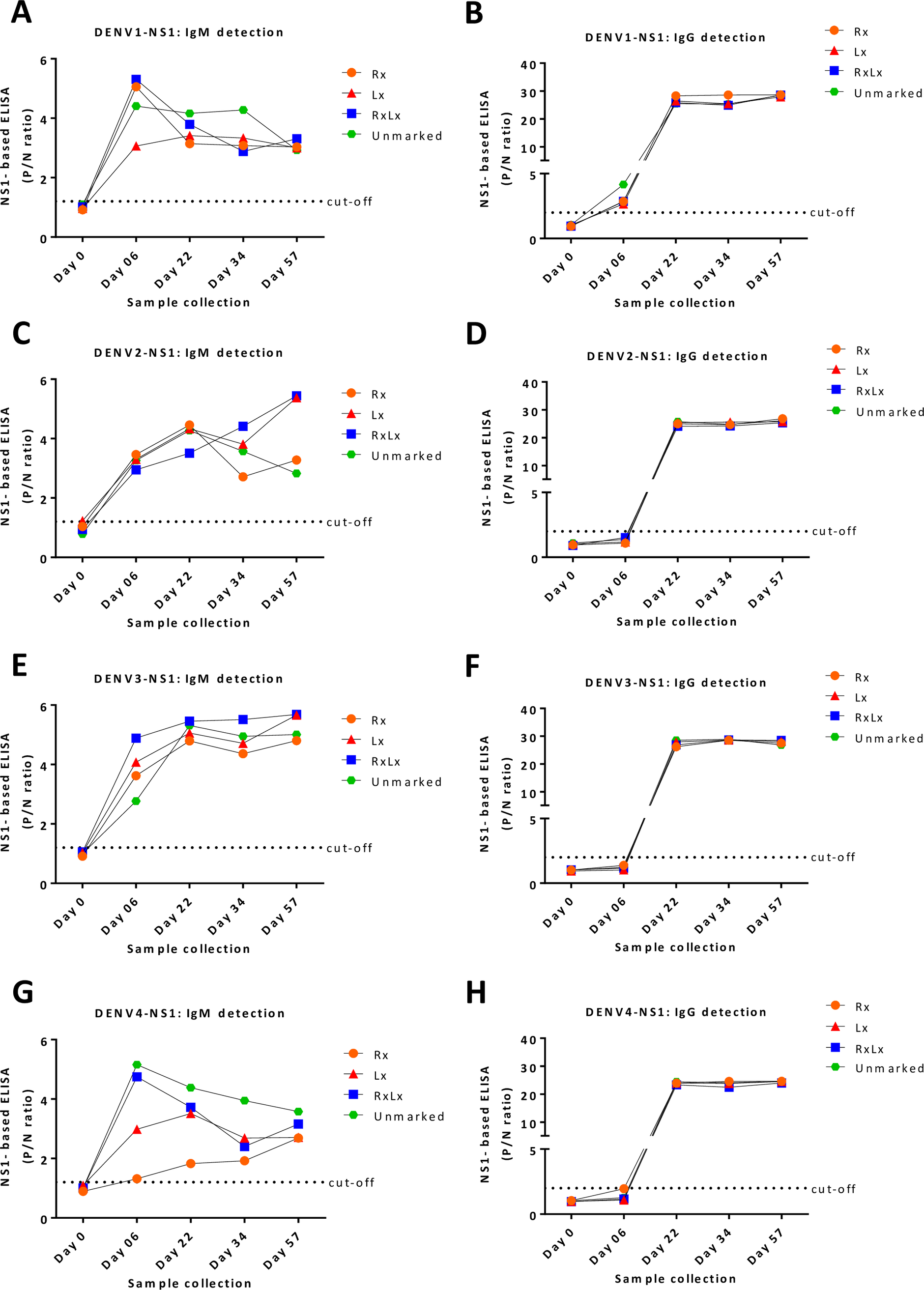

Supplement: S3 Fig — A to H) Detection of IgM/IgG antibodies from 4 different immunized mice with NS1 V5-tagged construct for each DENV serotype (DENV-1, DENV-2, DENV-3, and DENV-4). Plates were coated with purified DENV1-rNS1, DENV2-rNS1, DENV3-rNS1 and DENV4-rNS1 antigens for the detection of antibodies from mice immunized with each DENV serotype, respectively. * each ELISA result includes the average of two biological replicates. (TIF) [file pntd.0008039.s003.tif]

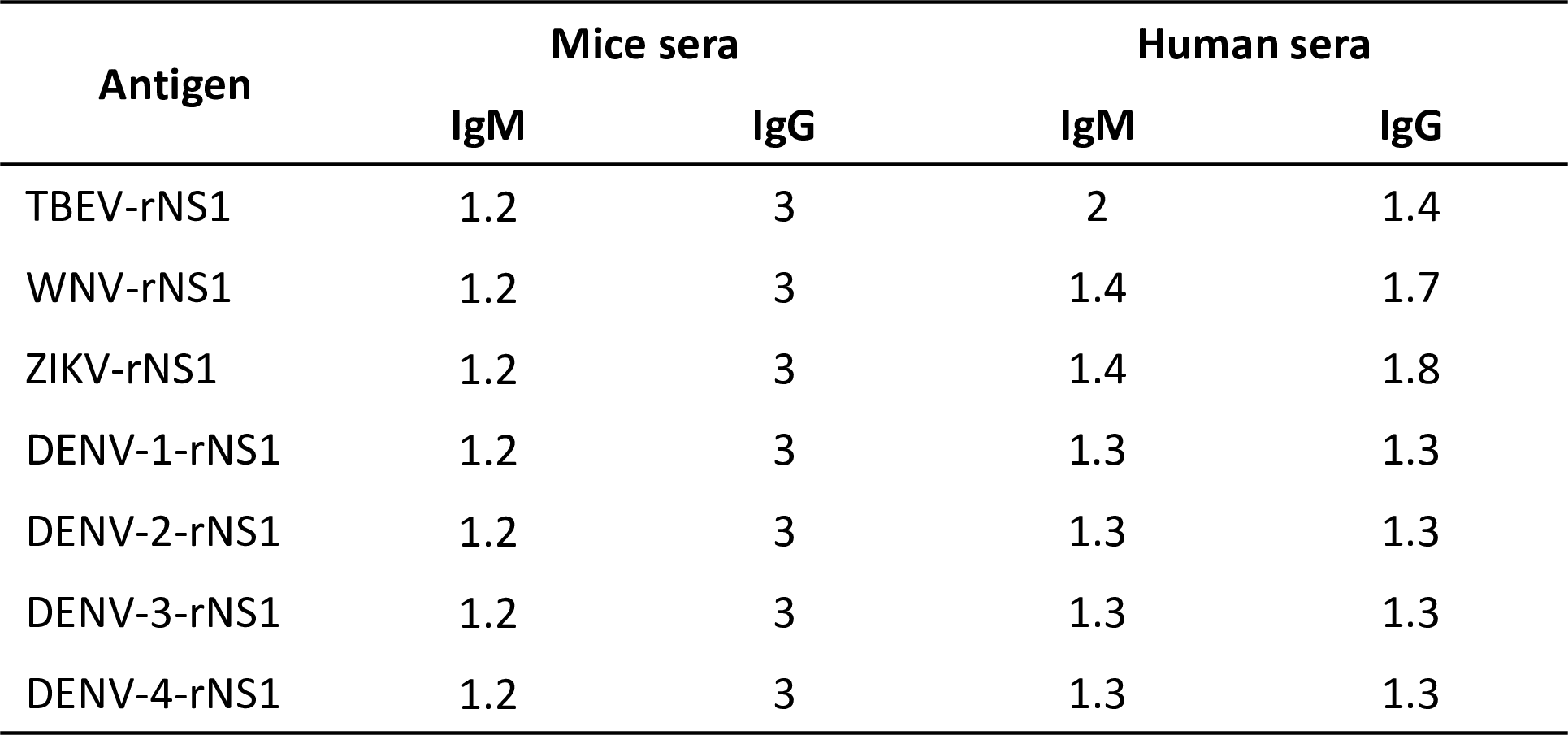

Supplement: S1 Table — P/N: positive to the negative ratio (OD450 of test specimen divided by the mean OD450 of negative control specimens). ROC: comparative receiver operating characteristic curve rNS1: recombinant non-structural protein 1 TBEV: Tick-borne encephalitis virus WNV: West Nile virus ZIKV: Zika virus DENV1-4: Dengue virus serotype 1, serotype 2, serotype 3 and serotype 4. (TIF) [file pntd.0008039.s004.tif]

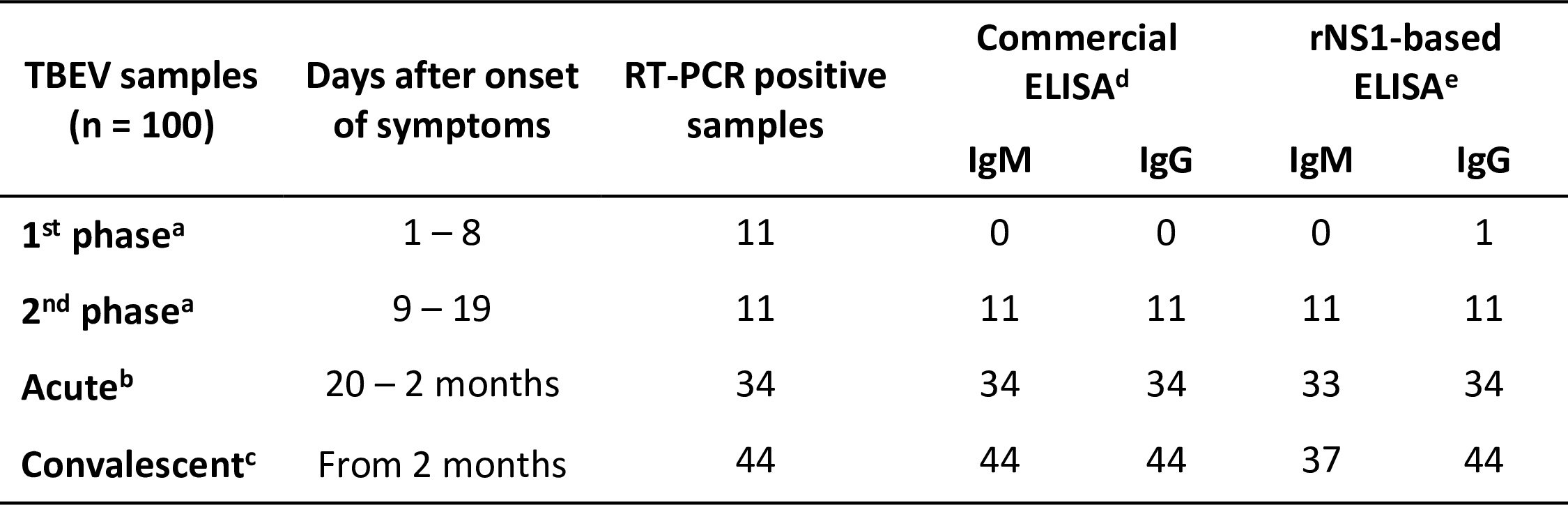

Supplement: S2 Table — TBEV: Tick-borne encephalitis virus ELISA: enzyme-linked immunosorbent assay rNS1: recombinant non-structural protein 1 a A total of 11 samples were tested for each group, first (1st) and second (2nd) phase of TBEV infection b A total of 34 samples were tested for the acute phase of TBEV infection c A total of 44 samples were tested for the convalescent phase of TBEV infection d plates were coated with inactivated TBEV E antigen, Enzygnost; Simens GmbH e plates were coated with recombinant non-structural protein 1 of TBEV. (TIF) [file pntd.0008039.s005.tif]

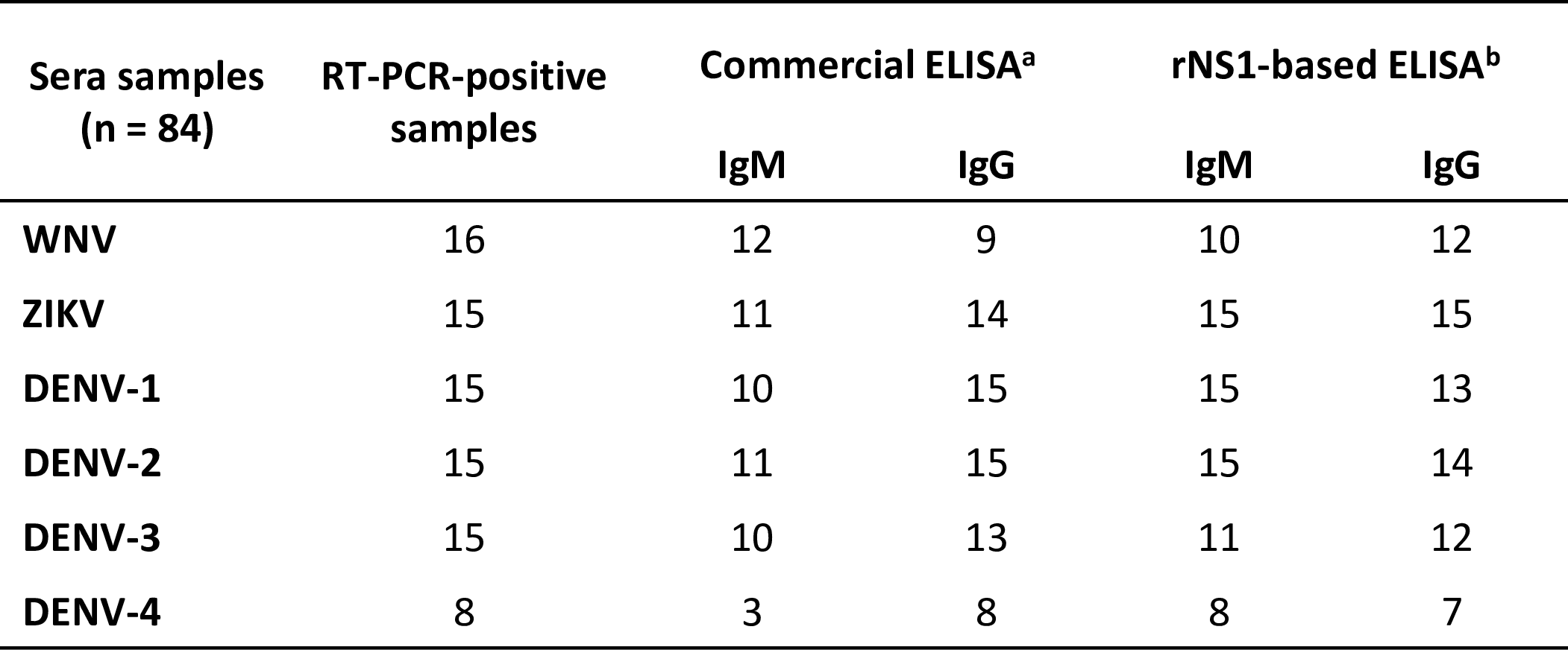

Supplement: S3 Table — WNV: West Nile virus ZIKV: Zika virus DENV1-4: Dengue virus serotype 1, serotype 2, serotype 3 and serotype 4 ELISA: enzyme-linked immunosorbent assay rNS1: recombinant non-structural protein 1 a IgM/IgG antibodies were detected by using commercial kits according to the manufacturer’s instructions for WNV (plates coated with recombinant WNV E antigen, Focus Diagnostics), ZIKV (plates coated with ZIKV recombinant NS1 antigen, Euroimmune; Labordiagnostika AG) and, DENV1-4 (plates coated with DENV type 2 E antigen, NovaTec, immunodiagnostic GmbH). b IgM/IgG antibodies were detected using purified rNS1 proteins of WNV, ZIKV and all DENV serotypes. (TIF) [file pntd.0008039.s006.tif]

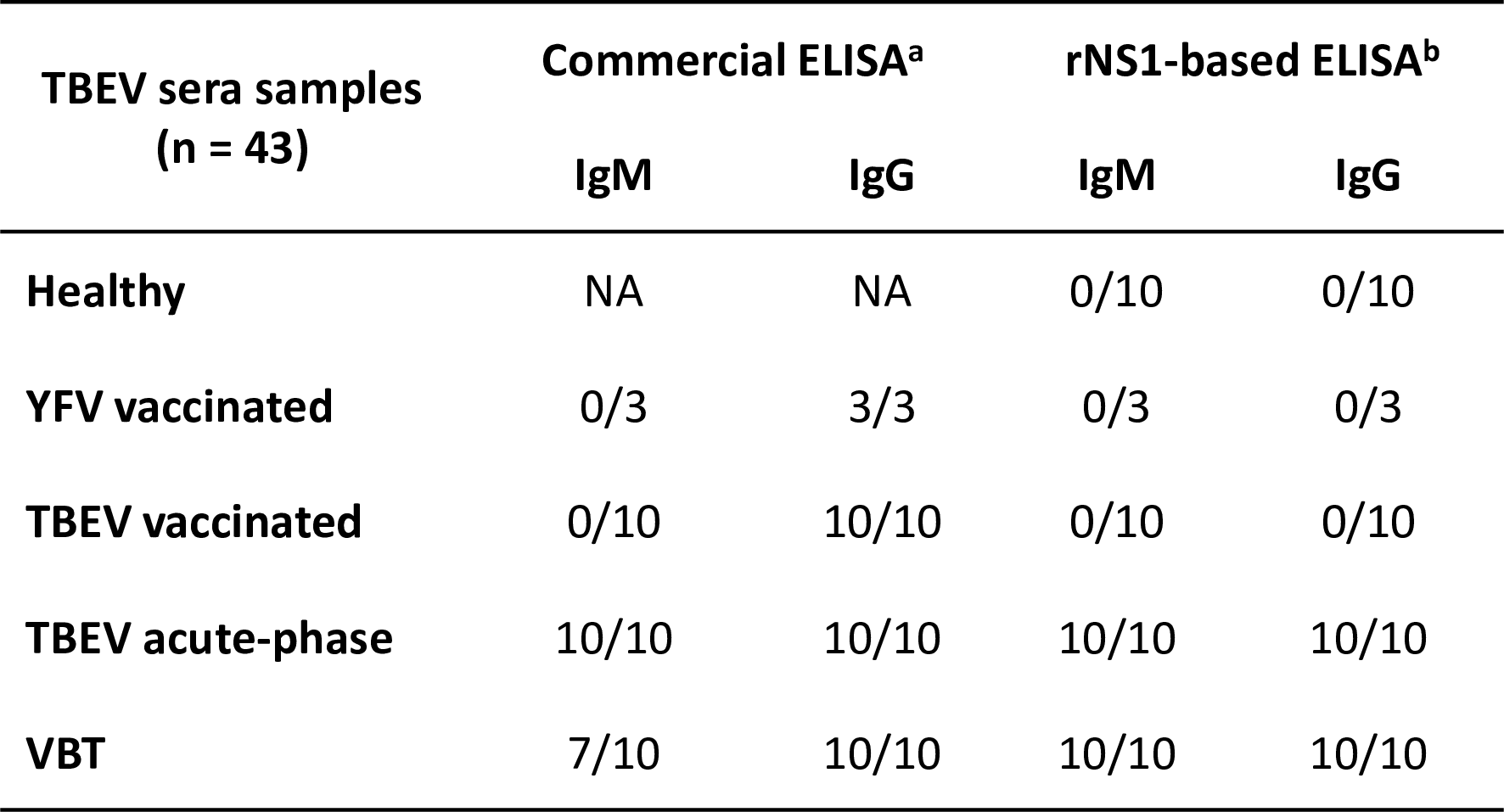

Supplement: S4 Table — TBEV: Tick-borne encephalitis virus ELISA: enzyme-linked immunosorbent assay rNS1: recombinant non-structural protein 1 VBT: vaccine breakthrough NA: non-available a IgM/IgG antibodies were detected by using a commercial kit according to the manufacturer’s instructions for TBEV (plates were coated with inactivated TBEV E antigen, Enzygnost; Simens GmbH b IgM/IgG antibodies were detected by rNS1-based ELISA coating the plates with the rNS1 protein of TBEV. (TIF) [file pntd.0008039.s007.tif]
