# Supplementary material for: Comparative specificity and sensitivity of NS1-based serological assays for the detection of flavivirus immune response
Source: PLoS Negl Trop Dis. 2020 Jan 29;14(1):e0008039. doi: 10.1371/journal.pntd.0008039 (PMC7010293; doi:10.1371/journal.pntd.0008039)
Supplement: Attachment — Submitted filename: REBUTTAL_ERICK_NS1_REV.pdf [file pntd.0008039.s008.pdf]

INTERNATIONAL CENTRE FOR GENETIC ENGINEERING AND BIOTECHNOLOGY
Padriciano 99, 34149 Trieste, Italy
http:www.icgeb.org

Alessandro Marcello, PhD
Head, Laboratory of Molecular Virology

Tel: +39-040-37571
Fax: +39-040-226555
E-mail: icgeb@icgeb.org

Sassan Asgari
Guest Editor

A. Desiree LaBeaud
Deputy Editor
PLOS Neglected Tropical Diseases

Trieste, 12th November 2019

Detailed response to the Reviewers' comments on manuscript '**Comparative specificity and sensitivity of NS1-based serological assays for the detection of Flavivirus immune response**' by Erick Mora Cardenas et al. for publication in PLOS NTD.

Reviewer's Responses to Questions

Key Review Criteria Required for Acceptance?
As you describe the new analyses required for acceptance, please consider the following:

Methods
-Are the objectives of the study clearly articulated with a clear testable hypothesis stated?
-Is the study design appropriate to address the stated objectives?
-Is the population clearly described and appropriate for the hypothesis being tested?
-Is the sample size sufficient to ensure adequate power to address the hypothesis being tested?
-Were correct statistical analysis used to support conclusions?
-Are there concerns about ethical or regulatory requirements being met?

Reviewer #1: Objectives and study design is very well described. Statistical analysis was appropriate. Human and animal ethic approvals are provided.

OK thanks

Reviewer #2: The study by Mora-Cardenas et al, evaluates the utility of antibodies to distinct flavi rNS1 proteins to be used detect seropositive response to distinct flavi viruses. The work is not especially novel as the use of NS1 specific antibodies to detect and identify exposure to different flavi viruses have been reported by multiple groups previously. In this study the authors specifically evaluated recombinant NS1 expressed in 293HEK cells from several flaviviruses including tick-borne encephalitis, west nile, zika and dengue virus (1-4). However it was somewhat surprising that JE and YF were not included. The initial analysis examined the purified proteins. It was well performed and demonstrated the successful expression of the mature glycosylated hexamer form in the culture supernatan

The reviewer is correct in pointing out that the use of NS1 antigen for detecting flavivirus antibodies has been described previously. However, our study provides (i) a scheme for NS1 antigen purification; (ii) comparative analysis of the ability of NS1 antigens to detect IgM/G responses from mice immunized with NS1 from different ticks and mosquitoes-derived flaviviruses; (iv) a comparative analysis of the IgM/G response to different flaviviruses in human samples by NS1-based ELISA and commercial ELISA assays. Therefore, this study is novel in providing a comprehensive parallel analysis of the use of NS1 as antigen for flavivirus IgM/G detection and in providing a strategy for its implementation starting from the purification of the antigens.
Concerning YFV and JEV antigens, we did apply the same strategy of codon-optimization and expression of the other antigens, but were not successful in their purification in the

amounts needed for the assays. Indeed, we observed that the expression/secretion of NS1 from different flaviviruses varies a lot, with certain antigens such as NS1 derived from ZIKV that could be purified in large amounts from the culture supernatant, while NS1 derived from DENV3 was extremely difficult to purify. These data, in addition to the lack of human samples from patients infected with YFV or JEV, discouraged us to proceed further. However, in one additional study, currently submitted for publication in a separate article, we were successful in purifying and testing Usutu virus (USUV) NS1. Usutu virus is of high importance locally as it has become endemic in Europe. Since only few assays are available commercially for the detection of USUV we focused on this antigen. The abstract of this manuscript is appended to this reply, full text is available if required. (*)

Reviewer #3: Mora-Cardenas et al have clearly articulated the goals of the presented study. I have some concerns over the generally small sample sizes for human samples for dengue Zika and WNV.

This study wanted to compare several NS1 antigens from different ticks and mosquitoes-derived flaviviruses for their ability to detect IgM/G responses (see above) rather than focusing on a single infection. Since the analysis was meant to be on multiple antigens in parallel, rather than one single antigen in depth, the number of samples was smaller for each ZIKV, DENV and WNV antigen, but relevant on the whole. In addition, we tested more samples for TBEV because of the importance of vaccine brake-through cases that are increasing and of great concern in Europe.
Follow up work with these NS1 antigens is currently being conducted on 200 sera from Nigeria, which is highly endemic for these infections. These data include also clinical, molecular and epidemiological data and will be submitted as a separate study.

Results
-Does the analysis presented match the analysis plan?
-Are the results clearly and completely presented?
-Are the figures (Tables, Images) of sufficient quality for clarity?

Reviewer #1: The results are very clearly presented and fulfill the objectives put forward by the researchers

OK thanks

Reviewer #2: There are some minor inconsistencies that will be shared in the comments to the authors. The results are generally clear. the figures could use some additional clarification or annotations.

OK thanks

Reviewer #3: The describe a NS1 expression from HEK293 cells however they fail to provide details of the transfection. This need to be detailed in the methods section. The authors state they have produced “fully antigenic native recombinant NS1 proteins….” Remove native from this statement. Furthermore, in figure 1 where authors perform western blot analysis the proteins are probed with anti-V5 or anti-His antibody not an anti-NS1 antibody. While it is highly likely these antigens are antigenically correct, to confirm this the recombinant NS1 proteins should interrogated with MAbs to each domain. The only data in the paper to suggest these antigens are antigenically similar to their native counter parts is the reactivity to human sera. Size exclusion chromatography should be performed to confirm the hexameric state of the expressed NS1 protein.
Likewise, more detail is required in the explanation of the endo H and PNGase F digestions, ie molecular weight shifts, presence of high mannose carbohydrates on endo H samples, not observed in WNV…. This needs more detail in the figure, the text and caption.
Figure 2 C and D Rx, Lx, RxLx are not described in detail to adequately inform the reader
While not critical to reproducing the results from the manuscript, authors have omitted all details of SDS-PAGE gel analysis in the methods

As the reviewer points out, it is highly likely that the 7 recombinant NS1 are antigenically similar to their native counterparts. Previous reports have shown that flavivirus NS1, either native or recombinant, is secreted as a lipoprotein hexamer in mammalian cells, which is very sensitive even to mild detergents. In this paper we provide evidence based on native PAGE analysis that the secreted NS1 antigens are composed of high oligomers consistent with the hexameric forms already described in literature. Consistently, more stable characteristic dimers of NS1 were observed in unheated/non-reducing SDS PAGE.

We also provide a glycosylation analysis for all 7 NS1 antigens purified from cells of human origin. This material was used for IgM/G detection in mice and human sera samples. Additional biochemical analysis could provide a better characterization for the secreted proteins from different flaviviruses, but would provide valuable additional information only if correlated to the antigenicity of the protein, particularly for the understanding of cross-reactivity. This would require work that is beyond the scope for this article. (**)

Methodology details have been added and text/figures modified according to suggestions.

Conclusions
-Are the conclusions supported by the data presented?
-Are the limitations of analysis clearly described?
-Do the authors discuss how these data can be helpful to advance our understanding of the topic under study?
-Is public health relevance addressed?

Reviewer #1: Mora-Cardenas et al present a straightforward study investigating the use of the NS1 protein as a diagnostic antigen for a range of flaviviruses. Although NS1 has been well investigated before, the wide range of different flaviviruses utilized in this work (including both mosquito and tick borne viruses) makes this a unique and valuable addition to the literature. In addition to validation of the antigens in mouse models, the researchers present data from a range of human clinical samples to support the use of recombinant NS1 for both IgM and IgG flavivirus serology.

OK thanks

Reviewer #2: The evaluation of specificity and sensitivity are somewhat limited due to the availability of well characterized human samples. Nonetheless the studies are in agreement with similar studies on the utility of NS1 proteins and the antibodies generated to them post infections to evaluate individuals serostatus to different flaviviruses.

See comments above on the number of samples.

Reviewer #3: The conclusions are generally supported by the data presented with the study. As stated above the small numbers of dengue, Zika and WNV is a weakness. The TBE results support the conclusions. The ability to differential between natural infections, vaccinations by NS1 serology is a clear beneficial outcome to the field.

See comments above on the number of samples.

Editorial and Data Presentation Modifications?
Use this section for editorial suggestions as well as relatively minor modifications of existing data that would enhance clarity. If the only modifications needed are minor and/or editorial, you may wish to recommend “Minor Revision” or “Accept”.

Reviewer #1: Abstract – capitalization on West Nile, Zika
OK
Page 2 line 7 – individuals

OK
Author summary. First sentence reword
OK
Page 5 line 5 - insert commas “, from mammalian cells,”
OK
Page 5 line 6 - remove "also"
OK
Page 6 line 19 and Fig 1. "non-denaturing" normally refers to without SDS. Suggest changing to unboiled or unheated to clarify. Could also describe the usual TBEV result as a SDS resistant hexameric species
OK, better clarified in the text
Page 6 line 30 – change "contain the N-linked glycans" to "are modified with N-linked glycans". The specific glycosylation sites are not confirmed, but the data can show that the proteins are being glycosylated at a whole protein level.
OK
Fig1 – no need for the domain labeling, this is inaccurate and not relevant for this work in any case
OK
Fig1 C – native gel analysis, not possible to confirm the oligomeric species – crosslinking and SDS-PAGE or alternatively size exclusion chromotography would have been better, perhaps worth adding a point to the results/discussion near the unusual TBEV result and possible future investigation/validation.
OK addressed in the text as suggested

Reviewer #2: Although this work is not especially novel, the characterization of the rNS1 proteins was well performed and might be of interest in comparison those available commercially and expressed in different systems used in other studies.

OK thanks

Reviewer #3: See results section for suggestion to improve clarity of figure 1 and potential additional experiments for the characterisation of NS1.

See comments above (*).

Summary and General Comments
Use this section to provide overall comments, discuss strengths/weaknesses of the study, novelty, significance, general execution and scholarship. You may also include additional comments for the author, including concerns about dual publication, research ethics, or publication ethics. If requesting major revision, please articulate the new experiments that are needed.

Reviewer #1: This is a straightforward study describing the use of recombinant NS1 as a flavivirus diagnostic antigen. The recombinant NS1 is validated biochemically and validated using murine immunization and serological analysis. The analysis of a range of different clinical samples with comparisons with commercial kits is a particular strong point of the study. Overall the experimental work well is designed and results and discussion clearly presented.

OK thanks.

Reviewer #2: The study by Mora-Cardenas et al, evaluates the utility of antibodies to distinct flavi rNS1 proteins to be used detect seropositive response to distinct flavi viruses. The work is not especially novel as the use of NS1 specific antibodies to detect and identify exposure to different flavi viruses have been reported by multiple groups previously. In this study the authors specifically evaluated recombinant NS1 expressed in 293HEK cells from several flaviviruses including tick-borne encephalitis, west nile, zika and dengue virus (1-4). However it was somewhat surprising that JE and YF were not included. The initial analysis examined the purified proteins. It was well performed and demonstrated the successful expression of the mature glycosylated hexamer form in the culture supernatant. Although NS1 proteins are

available commercially from a variety of sources, the conformation of these proteins (monomer, dimer and hexamer) is reported to vary greatly depending on the sources and expression system used. The authors might consider including some of these in the biochemical characterization to elucidate some of the potential differences.
The immunogenicity assessment of the proteins was evaluated mice following immunization with the expression vector (DNA) rather than the purified protein. It was unclear why this approach was performed as differences in expression could result in varying levels of protein expression. The authors also employed different bleed schedules for the mice receiving the dengue constructs compared to those receiving the TBE, WNE and Zika constructs. Lastly the authors examined the specificity of the rNS1 proteins in ELISA-based methods in comparison to commercial based assays. These assay were limited by the number of available samples and often focused on samples from individuals with confirmed exposure to that flavivirus and those from flavi naïve individuals. In these analyses the sensitivity and specificity were high. However the analysis did not fully take into account the potential endemic nature of multiple flaviviruses in a given region. Consequently the specificity of the responses to the distinct rNS1 proteins would benefit from evaluating the potential reactivity of each NS1 proteins with human serum samples from individuals exposed to different flaviviruses (TBE, WNE, Zika, Dengue, in addition to JE or YF).
Despite these limitations, the study builds on earlier reports implicating the potential utility of recombinant NS1 proteins to detect exposure to distinct flaviviruses.

Comments

- The authors did not rNS1 from YF or JE in this evaluation.

See comments above (**).

- P.5/Line 10-12: Unclear what is meant by "Limited to TBEV, for which a vaccine is available for humans, rNS1 serology has the ability to detect vaccine breakthrough cases of infection"-this methodology is limited to use for TBEV?.

The sentence has been rephrased, see revised text.

- Figure 1: Panels C,D, and E would benefit from inclusion of molecular weight markers

OK

- Panel C middle panels- indicates "non-denat/reducing" but text p.6/line 19 seems to indicate non denat/non reducing. Please clarify.

OK

- The Last panel- indicates native, would this be equivalent to "non-denat/non-reduced"? Inclusion of molecular markers would be helpful to evaluate "hex" in last panel.

OK

- Figure 2 Panel A- please label to indicate virus per lane and indicate conditions used (eg denat/reduced?)

OK

- Figure 2 Panels C-H: Please clarify which bleed (#day post immunization) was used for the analysis. Please also confirm whether mouse serum was evaluated against rNS1 proteins that were HIS or V5 tagged.

OK added in the text

- P.7/Line 2- Despite characterization of His tagged proteins, immunological / immunogenicity evaluations were performed with V5-tagged. Was there a rationale for this change?

In fact, V5-tagged rNS1 was used only for DNA immunization, while H6-tagged purified rNS1 protein was used throughout the work for ELISA coating for immunological / immunogenicity evaluations.

- P.7/Line 5. Immogenicity was evaluated in BALB/c mice following intradermal immunization with plasmid DNA constructs. Was there a reason this approach was utilized rather than performing the evaluation with the purified HIS or V5 proteins?

The two different tags V5/H6 were used in the mice experiments to avoid cross-reactivity of the immunized sera towards the tag. Instead of purifying each of the seven NS1 antigens for immunization we decided to go for the intradermal gene gun route. We also considered that the first exposure of NS1 to the immune system occurs at the level of mosquito/tick bite, which occurs intradermally. Thus, delivery of the antigen by this route could be more physiologic. (***)

- P7/Lines 6-27: The authors are comparing the IgM and IgG response to TBE, WNE, Zika and Den 1-4 NS1 proteins. Most proteins were evaluated following regimen of 1,15,22 and 29. With bleeds on 0,19, 26 and 46. However regimen was changed for Dengue NS1 and mice were

immunized on 14 days intervals 1,15,29?(Not 22?) and bleed on days 0,6,22, 34, 57). The difference in the regimens makes it difficult to compare the responses, especially IgM since the early timepoint was only evaluate for the Den NS1 proteins.
For DENV 1-4 we followed a slightly different scheme for DENV1-4 NS1 immunization since there is an increase in the number of reports showing that high levels of IgG can affect the detection of IgM antibodies. We were concerned about the possibility of higher cross-reactivity within the DENV 4 serotypes and this prompted us to add an extra bleed 5 days post immunization. This was clarified in the text.

• P7/Line 28: In the specificity testing, it is not clear which bleeds (day) are being evaluated. Please clarify. It would be expected that specificity would increase over time with some reduced specificity temporarily post boost.
OK added in the text

• Page 8/line 22- TBEV diagnosed cases- can you please clarify how these individuals were diagnosed or confirmed (e.g. PCR?).
TBEV cases were all RT PCR confirmed (page 9)

• General notes: Throughout the text- In many sections of the manuscript the text is not clear when referring to the rNS1-ELISA being evaluated at the time. (example P.9/Line 29-30, “Whereas, 10/16 IgM and 12/16 IgG positives were obtained by rNS1-ELISA”. The text would be easier to follow if the authors could specify the antigen source (eg WNE- rNS1-ELISA)
OK clarified

Reviewer #3: Mora-Cardenas et al describe the expression and perform limited characterisation of recombinant sNS1 from transfected HEK293 cells. Authors then go on to demonstrate the use of NS1 as a target antigen for IgM/IgG serological analysis of vaccinated mice and human samples. The main focus of the article is serological analysis of TBE vaccinated and infected patients with a small number of Zika, dengue and West Nile patients. In attempting to demonstrate the broad utility of NS1 serological analysis the limited number of patients sera tested for dengue, WNV and Zika is a weakness of the manuscript. Furthermore, with the potential importance to disease outcome associated with dengue IgM/IgG serology in terms of primary vs secondary no comment was made regarding the potential value of this assay at identifying primary or secondary dengue. If the authors have access to a larger bank of human samples the manuscript would benefit from an increased sample size.
See comments above on the number of samples.

Provide the calcium phosphate transfection method briefly.
OK, see modified methods

What was the rational for choosing DNA vaccination of mice, given the authors are efficiently expressing protein why not vaccinate with the NS1 antigen itself in ID infection?

See comment above (***)

Sincerely,

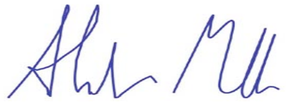

Alessandro Marcello, PhD
Head, Laboratory of Molecular Virology

PLOS Neglected Tropical Diseases

Comprehensive response to Usutu virus following first isolation in blood donors in the Friuli Venezia Giulia Region of Italy: development of recombinant NS1-based serology and sensitivity to antiviral drugs

--Manuscript Draft--

**Manuscript Number:**	PNTD-D-19-01776
**Full Title:**	Comprehensive response to Usutu virus following first isolation in blood donors in the Friuli Venezia Giulia Region of Italy: development of recombinant NS1-based serology and sensitivity to antiviral drugs
**Short Title:**	Usutu virus isolation from human in NE Italy: epidemiology, diagnostics and therapy
**Article Type:**	Research Article
**Keywords:**	Usutu virus; USUV; West Nile virus; WNV; diagnosis; Epidemiology; antiviral therapy; ELISA; NS1
**Corresponding Author:**	Alessandro Marcello<br>International Centre for Genetic Engineering and Biotechnology<br>Trieste, ITALY
**Corresponding Author Secondary Information:**	
**Corresponding Author's Institution:**	International Centre for Genetic Engineering and Biotechnology
**Corresponding Author's Secondary Institution:**	
**First Author:**	Ilaria Caracciolo
**First Author Secondary Information:**	
**Order of Authors:**	Ilaria Caracciolo
	Erick Mora-Cardenas
	Chiara Aloise
	Tea Carletti
	Ludovica Segat
	Maria Sole Burali
	Alexsia Chiarvesio
	Vivianna Totis
	Tatjana Avsic Zupanc
	Eloise Mastrangelo
	Giuseppe Manfroni
	Pierlanfranco D'Agaro
	Alessandro Marcello
**Order of Authors Secondary Information:**	
**Abstract:**	Surveillance of Usutu virus is crucial to prevent future outbreaks both in Europe and in other countries currently naïve to the infection, such as the Americas. This goal remains difficult to achieve, notably because of the lack of large-scale cohort studies and the absence of commercially available diagnostic reagents for USUV.<br>This work started with the first identification of USUV in a blood donor in the Friuli Venezia Giulia (FVG) Region in Northern-Eastern Italy, which is endemic for West Nile virus. Considering that only one IgG ELISA is commercially available, but none for IgM, a novel NS1 antigen based IgG/M ELISA has been developed. This assay tested successfully for the detection of Usutu virus in blood donors with the identification of a

Powered by Editorial Manager® and ProduXion Manager® from Aries Systems Corporation

second case of transmission and high levels of exposure. Furthermore, two pan-flavivirus antiviral drugs, that we previously characterized to be inhibitors of other flavivirus infectivity, were successfully tested for inhibition of Usutu virus with inhibitory concentrations in the low micromolar range.
To conclude, this work identifies North-Eastern Italy as endemic for Usutu virus with implications for the screening of transfusion blood. A novel NS1-based ELISA test has been implemented for the detection of IgM/G that will be of importance as a tool for the diagnosis and surveillance of Usutu virus infection. Finally, Usutu virus is shown to be sensitive to a class of promising pan-flavivirus drugs.

Powered by Editorial Manager® and ProduXion Manager® from Aries Systems Corporation
