# Supplementary material for: Comparative specificity and sensitivity of NS1-based serological assays for the detection of flavivirus immune response
Source: PLoS Negl Trop Dis. 2020 Jan 29;14(1):e0008039. doi: 10.1371/journal.pntd.0008039 (PMC7010293; doi:10.1371/journal.pntd.0008039)
Supplement: Attachment — Submitted filename: REBUTTAL_ERICK_NS1_REV2.doc [file pntd.0008039.s009.doc]

Padriciano 99, 34149 Trieste, Italy

http:www.icgeb.org

Alessandro Marcello, PhD

Head, Laboratory of Molecular Virology

Tel: +39-040-37571

Fax: +39-040-226555

E-mail: icgeb@icgeb.org

International Centre for Genetic Engineering and Biotechnology

Sassan Asgari

Guest Editor

A. Desiree LaBeaud

Deputy Editor

PLOS Neglected Tropical Diseases

Trieste, 20th December 2019

Response to the Reviewers’ comments on manuscript ‘**Comparative specificity and sensitivity of NS1-based serological assays for the detection of Flavivirus immune response**’ by Erick Mora Cardenas et al. for publication in PLOS NTD.

There are still two comments from Reviewer #2 to address:

Comment 1

*The last figure in the proofing package (following Supplemental Table 4) includes one labeled :”Striking figure”.  It is not clear what is being described in comparison to heat maps in Fig 2 (G,H), and no figure legend is provided*

The striking figure is a specific format of the journal and as required the legend is in the file StrikeImageCaption.docx

Comment 2

*Some remaining questions that could be addressed could include additional information around the specificity of the results when evaluating human samples.  Although data is provided regarding cross-reactivity of mouse serum following plasmid immunization, the specificity with human samples is not well characterized.  This is of particular importance with regards to samples from individuals post-acute/early convalescent infection, that are likely to have higher levels of broadly reactive antibodies. The limited number of samples is also recognized a potential weakness, but as long as the authors do not overstate conclusions, but discuss in terms of potential there are no concerns.*

We agree with Reviewer #2 that the discussion could be tempered down on the issue of specificity in human sera. Therefore the sentence below has been added to the Discussion.

*These preliminary data will need to be completed with a significant number of convalescent sera, likely to have broad cross-reactivity, and with sera from individuals exposed to different flaviviruses from endemic countries to provide a better picture of the specificity of the assay.*

We believe to have fully addressed all Reviewers’ comments, which contributed to a manuscript of better quality.

Sincerely,

Alessandro Marcello, PhD

Head, Laboratory of Molecular Virology
